# Functional connectivity gradients of the insula to different cerebral systems

**DOI:** 10.1002/hbm.26099

**Published:** 2022-10-07

**Authors:** Rui Wang, Fan Mo, Yuhao Shen, Yu Song, Huanhuan Cai, Jiajia Zhu

**Affiliations:** ^1^ Department of Radiology The First Affiliated Hospital of Anhui Medical University Hefei China; ^2^ Research Center of Clinical Medical Imaging, Anhui Province Hefei China; ^3^ Anhui Provincial Institute of Translational Medicine Hefei China

**Keywords:** resting‐state functional MRI, insula, hierarchical organization, functional connectivity gradients

## Abstract

The diverse functional roles of the insula may emerge from its heavy connectivity to an extensive network of cortical and subcortical areas. Despite several previous attempts to investigate the hierarchical organization of the insula by applying the recently developed gradient approach to insula‐to‐whole brain connectivity data, little is known about whether and how there is variability across connectivity gradients of the insula to different cerebral systems. Resting‐state functional MRI data from 793 healthy subjects were used to discover and validate functional connectivity gradients of the insula, which were computed based on its voxel‐wise functional connectivity profiles to distinct cerebral systems. We identified three primary patterns of functional connectivity gradients of the insula to distinct cerebral systems. The connectivity gradients to the higher‐order transmodal associative systems, including the prefrontal, posterior parietal, temporal cortices, and limbic lobule, showed a ventroanterior‐dorsal axis across the insula; those to the lower‐order unimodal primary systems, including the motor, somatosensory, and occipital cortices, displayed radiating transitions from dorsoanterior toward both ventroanterior and dorsoposterior parts of the insula; the connectivity gradient to the subcortical nuclei exhibited an organization along the anterior–posterior axis of the insula. Apart from complementing and extending previous literature on the heterogeneous connectivity patterns of insula subregions, the presented framework may offer ample opportunities to refine our understanding of the role of the insula in many brain disorders.

## Graphical abstract

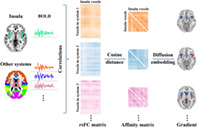

## 1 INTRODUCTION

The insula lies folded deep within the lateral sulcus of each hemisphere, hidden below parts of the frontal, parietal, and temporal lobes (Gogolla, 2017; Uddin et al., 2017). It is a uniquely located, cytoarchitectonically complex, and richly connected brain structure that typically acts as an integration hub involved in a wide variety of functions ranging from lower‐order sensorimotor processes to higher‐order cognition and emotion (Benarroch, 2019; Centanni et al., 2021; Gasquoine, 2014; Gogolla, 2017; Menon & Uddin, 2010; Molnar‐Szakacs & Uddin, 2022; Uddin et al., 2017). The diverse functional roles of the insula may emerge from its heavy connectivity to an extensive network of cortical and subcortical areas (Benarroch, 2019; Cauda et al., 2011; Cloutman et al., 2012; Gasquoine, 2014; Ghaziri et al., 2017; Gogolla, 2017; Taylor et al., 2009). Moreover, a large number of clinical neuroimaging studies have identified the insula as a core region affected across many psychiatric and neurological conditions including, but not limited to, autism (Lukito et al., 2020; Nomi et al., 2019; Uddin & Menon, 2009), schizophrenia (Qi et al., 2022; Sheffield et al., 2020; Xu et al., 2022; Zhu et al., 2015, 2017, 2018, 2020; Zhuo, Zhu, Qin, et al., 2017), depression (Kim & Han, 2021; Nord et al., 2021; Yu et al., 2020; Zhuo, Zhu, Wang, et al., 2017), addiction (Ghahremani et al., 2021; Perez Diaz et al., 2021; Qiu & Wang, 2021; Turel et al., 2021; Wei et al., 2017), anxiety disorders (Chavanne & Robinson, 2021; Cui et al., 2020; Feurer et al., 2021), Parkinson's disease (Carey et al., 2021; Pan et al., 2022; Tremblay et al., 2020), frontotemporal dementia (Gordon et al., 2016; Mandelli et al., 2016; Panman et al., 2019) and Alzheimer's disease (Jones et al., 2019; Liu et al., 2018; Nunez et al., 2020), highlighting its critical involvement in cross‐disorder neuropathological mechanisms. Collectively, these empirical insights from brain structural and functional data have exposed the insula as an integral component of behavior in various non‐disease and disease states, such that this brain structure has gained considerable attention in basic and clinical neuroscience.

On the basis of anatomy, connectivity, and functional contributions, the insula has been segmented into anywhere between 2 and 13 distinct subregions (Alcauter et al., 2015; Cauda et al., 2012; Centanni et al., 2021; Chang et al., 2013; Deen et al., 2011; Droutman et al., 2015; Faillenot et al., 2017; Ghaziri et al., 2018; Glasser et al., 2016; Kurth et al., 2010; Uddin et al., 2014; Uddin et al., 2017; Wysiadecki et al., 2018). At the simplest level, the insula is divided into anterior and posterior lobules by the central insular sulcus, with the anterior connected more to frontal and limbic areas and thus associated more with cognition and emotion, and the posterior connected more to motor and sensory areas and thus linked more with sensorimotor processes (Centanni et al., 2021; Faillenot et al., 2017; Uddin et al., 2017; Wysiadecki et al., 2018). Based on cytoarchitectonic analyses of the presence and density of cortical granular cell layer 4, the insula can be parsed into posterior granular, intermediate dysgranular, and anterior agranular subdivisions (Benarroch, 2019; Nieuwenhuys, 2012). Recently, more fine‐grained segments of the insula have been obtained by using functional and structural connectivity‐based parcellation techniques (Fan et al., 2016; Glasser et al., 2016; Nomi et al., 2016). It is noteworthy that most of these prior efforts have used data‐driven segmentation and clustering algorithms to parcellate the insula into subregions, i.e., insula voxels that share similar features (e.g., cytoarchitecture or connectivity patterns) were clustered and each cluster is assumed to represent a putative insula subregion. Given the discordance in the number of insula subregions reported between earlier studies, no consensus has been reached yet on how many clusters comprise the insula (Cauda & Vercelli, 2013). Instead, one may argue that topographic heterogeneity in macroscale insula features may be parsimoniously characterized as a continuum of gradual change (Huntenburg et al., 2018), rather than with discrete and independent subregions.

Diverse and convergent evidence demonstrates the existence of hierarchical organization in multiscale brain structures, which is reflected in structure, function, connectivity, and gene expression (Bajada et al., 2017; Gomez et al., 2019; Guell et al., 2018; Huntenburg et al., 2017; Huntenburg et al., 2018; Kharabian Masouleh et al., 2020; Margulies et al., 2016; Marquand et al., 2017; Paquola et al., 2019; Shine et al., 2019; Vogel et al., 2020; Vos de Wael et al., 2018; Wagstyl et al., 2015; Yang et al., 2020). A recently proposed gradient approach has provided a critical framework for the characterization of brain hierarchical organization, i.e., applying dimensionality reduction techniques to high‐dimensional brain features to obtain a parsimonious set of principal components that delineate continuous transitions of feature patterns across brain regions, referred to as gradients (Bajada et al., 2020; Hong et al., 2020; Vos de Wael et al., 2020). Several previous attempts, working within this framework, have been made to investigate the hierarchical principle of macroscale organization in the insula. For example, leveraging myelin‐sensitive magnetic resonance imaging (MRI), Royer and colleagues found two myeloarchitecture gradients in the human insula, one running from ventral anterior to posterior banks and one radiating from dorsal anterior toward both ventral anterior and posterior subregions (Royer et al., 2020). Taking advantage of quantitative modeling of multi‐shell diffusion MRI, Menon et al. identified insula microstructure gradients along its anterior–posterior and dorsal‐ventral axes (Menon et al., 2020). Employing a combination of diffusion tensor imaging and probabilistic white matter tractography, Cerliani et al. revealed a rostrocaudal trajectory of anatomical connectivity variation in the human insula (Cerliani et al., 2012). A recent functional MRI (fMRI) study demonstrated that resting‐state functional connectivity (rsFC) diversity of the insula could be most parsimoniously modeled as continuum of gradual change from dorsal‐posterior to ventral‐anterior, and inter‐individual variation in this continuum could explain significant variation in behavior (Tian & Zalesky, 2018). However, these studies constructed connectivity gradients of the insula based on its anatomical or functional connectivity to the whole brain, leaving open the question of whether and how there is variability across connectivity gradients of the insula to different cerebral systems.

To address this question, we used resting‐state fMRI data from 793 healthy subjects to discover and validate functional connectivity gradients of the insula, which were computed based on its voxel‐wise rsFC profiles to distinct cerebral systems. We hypothesized that functional connectivity gradients of the insula to cerebral systems with homogeneous features (e.g., lower‐order unimodal systems) would show similar patterns, which would differ from those to systems with heterogeneous characteristics (e.g., higher‐order transmodal systems).

## 2 MATERIALS AND METHODS

### 2.1 Participants

The study comprised a discovery dataset and two independent cross‐scanner, cross‐race validation datasets. The discovery participants were a sample of healthy, right‐handed Chinese Han adults, enrolled from the local universities and community via poster advertisements. Exclusion criteria included neuropsychiatric or severe somatic disorder, a history of head injury with consciousness loss, MRI contraindications, and a family history of psychiatric disease among first‐degree relatives. This study was approved by the ethics committee of The First Affiliated Hospital of Anhui Medical University. Written informed consent was obtained from all participants after they had been given a complete description of the study. The validation samples were derived from two publicly available datasets: Consortium for Neuropsychiatric Phenomics (CNP, https://openneuro.org/datasets/ds000030/versions/1.0.0) (Poldrack et al., 2016) and Southwest University Adult Lifespan Dataset (SALD, https://doi.org/10.15387/fcp_indi.sald) (Wei et al., 2018). Notably, we solely selected the healthy adults from the cross‐disorder CNP dataset. For the CNP dataset, healthy participants were excluded if they had lifetime diagnoses of psychiatric disorders, left handedness, pregnancy, or other contraindications to scanning; for the SALD dataset, the exclusion criteria included MRI contraindications, current psychiatric or neurological disorders, use of psychiatric drugs within 3 months, pregnancy, or a history of head trauma. Full details regarding the two validation samples (e.g., ethics, informed consent, inclusion, and exclusion criteria, among others) have been described in the previous studies (Poldrack et al., 2016; Wei et al., 2018). To exclude the potential influence of neurodevelopment and neurodegeneration, all the participants were restricted to an age range of 18–60 years. In addition, participants with poor image quality or excessive head motion during scanning were excluded. This brought the final samples used in the current study to 361 (183 females) in the discovery dataset, 103 (47 females) in the CNP dataset, and 329 (207 females) in the SALD dataset. Demographic data of the three datasets are detailed in Table S1 in the Supplementary materials.

### 2.2 MRI data acquisition

Resting‐state functional MRI data of the discovery sample were acquired using the 3.0‐Tesla General Electric Discovery MR750w scanner, and those of the validation samples were obtained using the 3.0‐Tesla Siemens Trio scanners. Details of the acquisition parameters for the three datasets are shown in Table S2 in the Supplementary materials.

### 2.3 Resting‐state fMRI data preprocessing

Resting‐state blood‐oxygen‐level‐dependent (BOLD) data were preprocessed using Statistical Parametric Mapping (SPM12, http://www.fil.ion.ucl.ac.uk/spm) and Data Processing & Analysis for Brain Imaging (DPABI, http://rfmri.org/dpabi) (Yan et al., 2016). The first several brain volumes (discovery: 10, CNP: 5, SALD: 10) for each participant were discarded to allow the signal to reach equilibrium and the participants to adapt to scanning noise. The remaining volumes were corrected for the acquisition time delay between slices. Then, realignment was performed to correct the motion between volumes. Head motion parameters were computed by estimating the translation in each direction and the angular rotation on each axis for each volume. All participants' BOLD data were within the defined motion thresholds (i.e., maximum translational or rotational motion parameters <2.0 mm or 2.0°). We also calculated frame‐wise displacement (FD), which indexes the volume‐to‐volume changes in head position. Several nuisance covariates (the linear drift, the estimated motion parameters based on the Friston‐24 model, the spike volumes with FD >0.5 mm, the white matter signal, and the cerebrospinal fluid signal) were regressed out from the data. Next, the datasets were band‐pass filtered using a frequency range of 0.01 to 0.1 Hz. In the normalization step, individual high‐resolution structural images were firstly co‐registered with the mean functional images; then the transformed structural images were segmented and normalized to the Montreal Neurological Institute (MNI) space using the diffeomorphic anatomical registration through the exponentiated Lie algebra (DARTEL) technique (Ashburner, 2007). Finally, each filtered functional volume was spatially normalized to the MNI space using the deformation parameters estimated during the above step and resampled into a 3‐mm cubic voxel.

### 2.4 Definition of the insula and other cerebral systems

**FIGURE 1:**
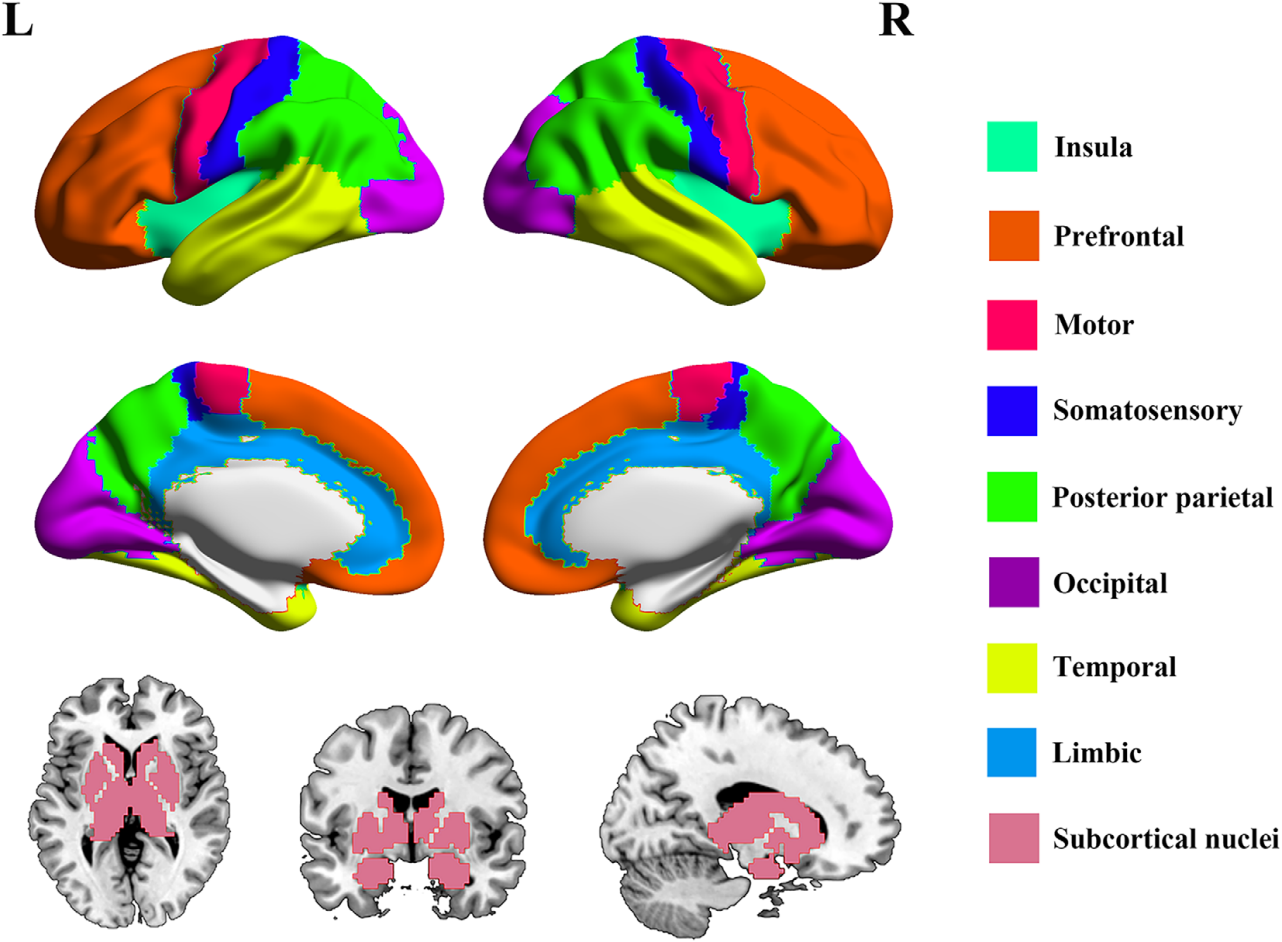
Illustration of the insula and other cerebral systems. Abbreviations: L, left; R, right

The Human Brainnetome Atlas (Fan et al., 2016) is a new brain atlas constructed using a connectivity‐based parcellation framework, such that its brain regions are obtained based on their specific anatomical and functional connectivity patterns. We adopted this atlas to define the insula (965 voxels) and other eight cerebral systems. Specifically, the prefrontal cortex (10,667 voxels) comprises the superior frontal, middle frontal, inferior frontal, and orbitofrontal gyri; the motor cortex (2492 voxels) consists of the precentral gyrus and anterior portion of the paracentral lobule; the somatosensory cortex (1962 voxels) includes the postcentral gyrus and posterior portion of the paracentral lobule; the posterior parietal cortex (7076 voxels) comprises the superior parietal lobule, inferior parietal lobule, and precuneus; the occipital cortex (5157 voxels) consists of the medioventral occipital and lateral occipital cortices; the temporal cortex (8209 voxels) includes the superior temporal, middle temporal, inferior temporal, fusiform, and parahippocampal gyri as well as posterior superior temporal sulcus; the limbic lobule (1661 voxels) refers to the cingulate gyrus; the subcortical nuclei (3234 voxels) comprises the amygdala, hippocampus, basal ganglia, and thalamus (Figure 1 and Table S3 in the Supplementary materials).

### 2.5 Calculation of functional connectivity gradients

**FIGURE 2:**
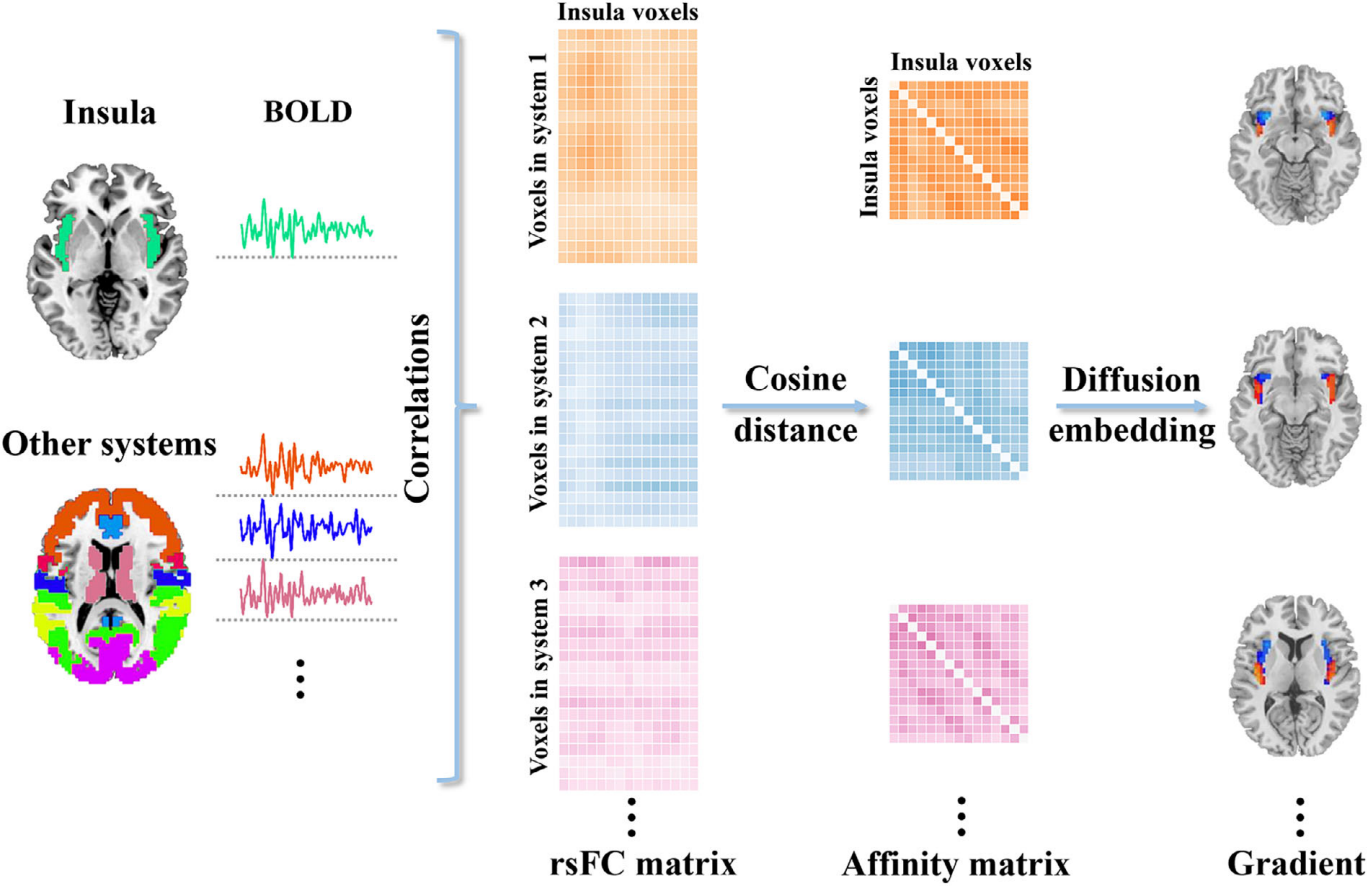
Calculating procedure of functional connectivity gradients of the insula. For each cerebral system, a voxel‐wise insula‐to‐cerebral system rsFC matrix was generated by calculating Pearson's correlation coefficients between BOLD time courses of each voxel within the insula and each voxel within that cerebral system. For each resultant rsFC matrix, similarity between all pairs of rows was calculated using cosine distance, yielding a positive symmetric affinity matrix reflecting similarity of rsFC profiles between each pair of voxels within the insula. Functional connectivity gradients were then calculated using diffusion map embedding. For each cerebral system, we focused on the first gradient that explained the greatest connectivity variance and a gradient value was assigned to each voxel within the insula, generating an insula gradient map to visualize the gradient topography. Abbreviations: BOLD, blood‐oxygen‐level‐dependent; rsFC, resting‐state functional connectivity

A schematic overview of the calculating procedure of functional connectivity gradients of the insula is presented in Figure 2. Functional connectivity gradients were calculated based on rsFC of the insula to the other eight cerebral systems. First, the preprocessed BOLD images were concatenated across all subjects after standardization using *z*‐scores, yielding group‐level BOLD time courses. Second, for each cerebral system, a voxel‐wise insula‐to‐cerebral system rsFC matrix was generated by calculating Pearson's correlation coefficients between group‐level BOLD time courses of each voxel within the insula and each voxel within that cerebral system. The resultant eight rsFC matrices (insula‐to‐prefrontal cortex: 965 × 10,667; insula‐to‐motor cortex: 965 × 2492; insula‐to‐somatosensory cortex: 965 × 1962; insula‐to‐posterior parietal cortex: 965 × 7076; insula‐to‐occipital cortex: 965 × 5157; insula‐to‐temporal cortex: 965 × 8209; insula‐to‐limbic lobule: 965 × 1661; insula‐to‐subcortical nuclei: 965 × 3234) were Fisher's *Z*‐transformed to improve normality. Then, for each row in the rsFC matrix, the top 10% rsFC values were retained, whereas all others were set to zeros (Margulies et al., 2016; Yang et al., 2020). Third, similarity between all pairs of rows was calculated using cosine distance, yielding a positive symmetric affinity matrix (965 × 965) reflecting similarity of rsFC profiles between each pair of voxels within the insula.

We calculated functional connectivity gradients using diffusion map embedding (Coifman et al., 2005; Guell et al., 2018; Margulies et al., 2016), a nonlinear dimensionality reduction technique that can recover a low‐dimensional embedding from high‐dimensional connectivity data. In the embedding space, voxels that are strongly connected by either many connections or few very strong connections are close, whereas voxels with little or no connections are far apart. Compared with other non‐linear dimensionality reduction algorithms, diffusion map embedding is relatively robust to noise, computationally inexpensive, and provides a stable representation of connections (Lafon & Lee, 2016). By applying this algorithm to each affinity matrix, we identified multiple low‐dimensional gradients accounting for connectivity variance in descending order. For each gradient, a gradient value (normalized using *z*‐scores) was assigned to each voxel within the insula, resulting in an insula gradient map to visualize macroscale continuous transitions in overall connectivity patterns, i.e., the gradient topography. We focused on the first gradient (i.e., the principal gradient) that explained the greatest variance in connectivity. Notably, the diffusion map embedding is controlled by a single parameter *α*, which controls the influence of the density of sampling points on the underlying manifold (*α* = 0, maximal influence; *α* = 1, no influence). Following previous studies (Dong et al., 2020; Guell et al., 2018; Hong et al., 2019; Margulies et al., 2016; Yang et al., 2020), we set *α* = 0.5 that is considered well‐suited for the analysis of brain connectivity data.

### 2.6 Validation analyses

Several validation analyses were conducted to verify the robustness of our results. First, our main analyses were performed in the discovery dataset. To exclude the influence of samples, we repeated the above‐described analyses in two independent cross‐race, cross‐scanner validation datasets (CNP and SALD). Second, before calculating the affinity matrix, we retained the top 10% rsFC values per row in the rsFC matrix. To examine the effect of threshold selections, we re‐ran our analysis with use of two other thresholds (top 20% and 30%). Finally, global signal regression (GSR) has been a controversial step in the preprocessing of resting‐state fMRI data (Murphy & Fox, 2017). To assess its potential impact, we re‐computed the rsFC matrix based on BOLD data with GSR and then repeated the functional connectivity gradient analyses.

## 3 RESULTS

### 3.1 Functional connectivity gradients of the insula

**FIGURE 3:**
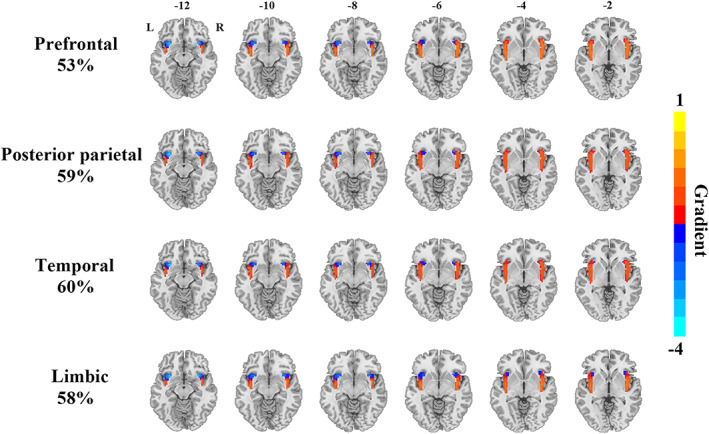
Functional connectivity gradients of the insula to the higher‐order transmodal associative systems including the prefrontal, posterior parietal, temporal cortices and limbic lobule. The percentages represent connectivity variance explained by the corresponding gradients. Abbreviations: L, left; R, right

**FIGURE 4:**
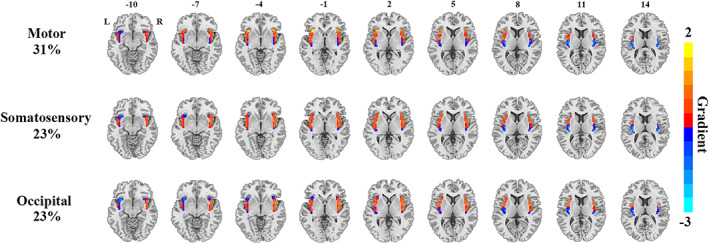
Functional connectivity gradients of the insula to the lower‐order unimodal primary systems including the motor, somatosensory, and occipital cortices. The percentages represent connectivity variance explained by the corresponding gradients. Abbreviations: L, left; R, right

**FIGURE 5:**
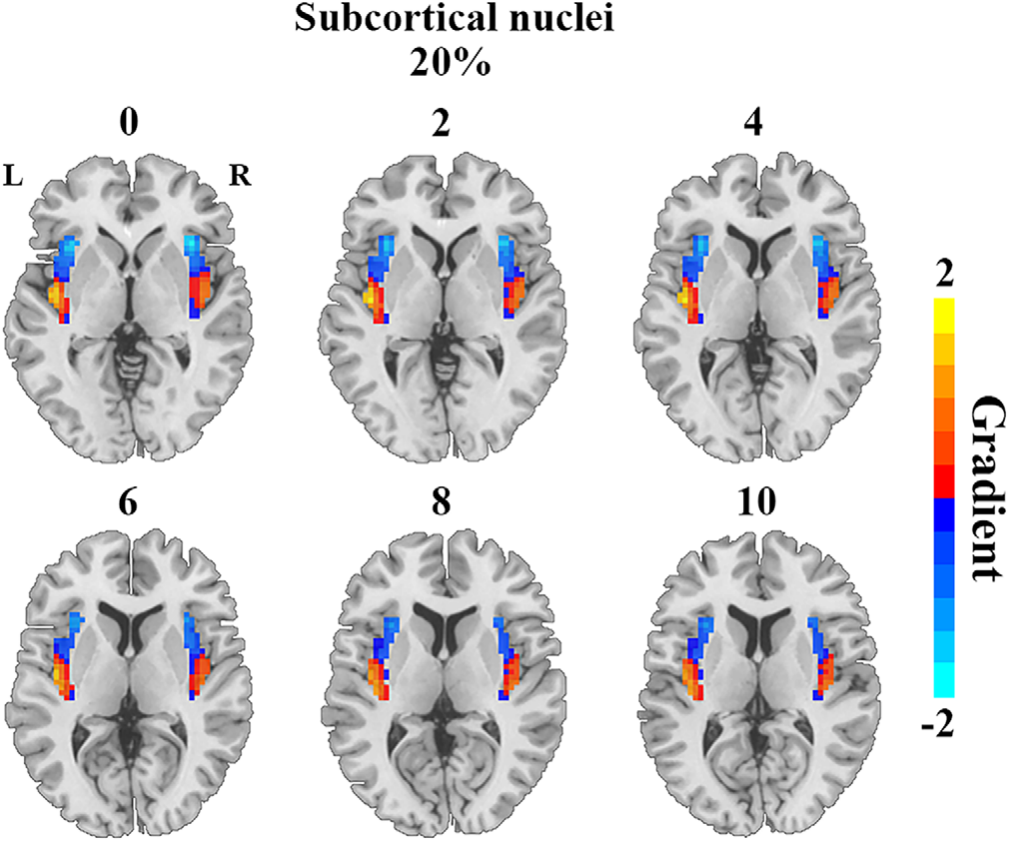
Functional connectivity gradient of the insula to the subcortical nuclei. The percentage represents connectivity variance explained by the gradient. Abbreviations: L, left; R, right

Functional connectivity gradients of the insula presented distinct topographies along with different degrees of explained connectivity variance across cerebral systems, which could be summarized as three primary patterns. The first pattern was observed for the functional connectivity gradients of the insula to the higher‐order transmodal associative systems including the prefrontal, posterior parietal, temporal cortices, and limbic lobule, which accounted for a high degree of connectivity variance (53%–60%). This pattern showed a ventroanterior‐dorsal axis across the insula, characterized by a gradual change from ventroanterior to dorsal portion (Figure 3). The second pattern was found for the functional connectivity gradients of the insula to the lower‐order unimodal primary systems including the motor, somatosensory, and occipital cortices, which explained a moderate degree of connectivity variance (23%–31%). This pattern displayed radiating transitions from dorsoanterior toward both ventroanterior and dorsoposterior parts of the insula (Figure 4). The third pattern was seen for the functional connectivity gradient of the insula to the subcortical nuclei, which accounted for a relatively low degree of connectivity variance (20%). This pattern exhibited an organization along the anterior–posterior axis, characterized by a gradual change from anterior to posterior portion of the insula (Figure 5). Scree plots showing the connectivity variance explained by the gradients of the insula to the eight cerebral systems are provided in Figure S1 in the Supplementary materials.

### 3.2 Validation analyses

The results of validation analyses supported the robustness of our findings. First, the insula functional connectivity gradients derived from two independent cross‐race, cross‐scanner validation datasets (CNP and SALD) were largely consistent with those from the discovery dataset (Figures S2–S4 in the Supplementary materials), indicating no influence of samples. Second, when applying two other thresholds (top 20% and 30%) to the rsFC matrix, we found that explained connectivity variance and topographies of the insula functional connectivity gradients were nearly identical to those using the threshold of top 10% (Figures S5–S7 in the Supplementary materials), suggesting no effect of threshold selections. Finally, analyzing BOLD data with GSR yielded insula functional connectivity gradients similar to those in our main analysis of BOLD data without GSR, with exception of the insula‐to‐motor cortex and insula‐to‐subcortical nuclei gradients (Figures S8–S10 in the Supplementary materials).

## 4 DISCUSSION

The insula has long been considered a key cortical hub with widespread connectivity to a rich range of cortical and subcortical regions serving sensorimotor, emotional, and cognitive processes, which gives rise to its diverse functional roles (Benarroch, 2019; Centanni et al., 2021; Chen et al., 2018; Gasquoine, 2014; Gogolla, 2017; He et al., 2019; Menon & Uddin, 2010; Molnar‐Szakacs & Uddin, 2022; Uddin et al., 2017). For instance, it is well established that the insula has a wide array of connections with the prefrontal, motor, somatosensory, posterior parietal, occipital, and temporal cortices as well as limbic lobule and subcortical nuclei (Benarroch, 2019; Cauda et al., 2011; Cloutman et al., 2012; Gasquoine, 2014; Ghaziri et al., 2017; Gogolla, 2017; Taylor et al., 2009). From a network perspective, the insula functions as a causal outflow hub within the salience network that permits controls of network dynamics through direct and indirect links to important nodes within major networks including the central executive, default mode, dorsal attention, ventral attention, sensorimotor, visual, and auditory networks (Cauda et al., 2011; Centanni et al., 2021; Menon & Uddin, 2010; Molnar‐Szakacs & Uddin, 2022). Crucially, there is solid evidence that different insula subregions show distinct structural and functional connectivity patterns (Alcauter et al., 2015; Cauda et al., 2012; Centanni et al., 2021; Chang et al., 2013; Deen et al., 2011; Fan et al., 2016; Ghaziri et al., 2017; Ghaziri et al., 2018; Glasser et al., 2016; Nomi et al., 2016). Altogether, this previous literature has led to some speculation that there may be heterogeneous hierarchical organizations within the insula reflecting its connectivity profiles to different cerebral systems.

The high‐dimensionality of brain connectivity data lies in the fact that each brain location has more than one connection. In this context, dimensionality reduction techniques are required to extract intelligible information from such high‐dimensional connectivity data. One commonly used method is to group brain locations into larger parcels based on connectivity similarity, i.e., clustering‐based parcellation of insula subregions. Nevertheless, treating subregions as discrete and independent entities cannot characterize more gradual changes and overarching spatial relationships (Jbabdi et al., 2013). Instead, the recently developed gradient approach finds the main axes of variance in the data through decomposition or embedding algorithms, and replaces the original high dimensions of connectivity data with a more parsimonious set of new dimensions (i.e., connectivity gradients) that account for most of the connectivity variance. Each gradient is a continuous representation of one aspect of insula connectivity hierarchy, and each brain location can be quantified by a value reflective of where it falls along this gradient. Extensive research has established that the spatial arrangement of brain locations along these connectivity gradients is not arbitrary, but rather a consequence of developmental mechanisms shaped through evolutionary selection (Huntenburg et al., 2018). Combined, a gradient‐based rather than cluster‐based conceptualization may facilitate a more thorough characterization of the hierarchical organization of insula connectivity.

By applying Laplacian eigenmaps to anatomical connectivity data derived from diffusion tensor imaging and probabilistic white matter tractography, Cerliani et al. revealed a rostrocaudal trajectory of anatomical connectivity variability ranging from anterior dorsal and ventral to dorsal caudal part of the insula (Cerliani et al., 2012). Likewise, application of Laplacian eigenmaps to functional connectivity data from resting‐state fMRI revealed that rsFC diversity across the insula's topography could be parsimoniously modeled as continuum of gradual change from dorsal‐posterior to ventral‐anterior; moreover, inter‐individual variation in insula functional connectivity gradients could account for inter‐individual difference in behavior (Tian & Zalesky, 2018), and insula functional connectivity gradients were altered in individuals with schizophrenia and related to clinical symptoms of this disorder (Tian et al., 2019). Despite these prior investigations demonstrating the potential usefulness of the gradient approach in delineating the hierarchical organization of insula connectivity in health and disease, their gradients were constructed based on insula‐to‐whole brain connectivity data, which may overlook the heterogeneity in connectivity profiles of the insula to different cerebral systems.

The current study opens new perspectives by being the first to examine functional connectivity gradients of the insula to distinct cerebral systems, and identified three primary patterns of connectivity gradients. In agreement with our expectations, the functional connectivity gradients to the higher‐order transmodal associative systems, including the prefrontal, posterior parietal, temporal cortices and limbic lobule, showed a ventroanterior‐dorsal axis across the insula; those to the lower‐order unimodal primary systems, including the motor, somatosensory, and occipital cortices, displayed radiating transitions from dorsoanterior toward both ventroanterior and dorsoposterior parts of the insula; the functional connectivity gradient to the subcortical nuclei exhibited an organization along the anterior–posterior axis of the insula. The observed topographies of insula connectivity gradients at the system level are partially consistent with those based on insula‐to‐whole brain connectivity reported in prior studies (Cerliani et al., 2012; Tian et al., 2019; Tian & Zalesky, 2018), suggesting that the latter likely involve some mixture of the former and thus may obscure specificity. More importantly, our data complement and extend previous literature on the heterogeneous connectivity patterns of insula subregions (Alcauter et al., 2015; Cauda et al., 2012; Centanni et al., 2021; Chang et al., 2013; Deen et al., 2011; Fan et al., 2016; Ghaziri et al., 2017, 2018; Glasser et al., 2016; Nomi et al., 2016; Zhao et al., 2022), by demonstrating that the connectivity heterogeneity of the insula can also be captured by its topographic organizations.

This study has several limitations that should be addressed in future work. First, to obtain more stable and reliable results, functional connectivity gradients of the insula were calculated at the group level rather than at the individual level, which may obscure meaningful inter‐individual differences. Second, we focused our analyses on the first gradient that accounted for the greatest variance in connectivity. Nevertheless, some physiologically significant gradients with smaller explained variance may be overlooked, hampering the possibility to achieve a more fruitful characterization of the insula functional connectivity hierarchy. Third, the cerebral systems were defined using the Human Brainnetome Atlas. Some data‐driven approaches, e.g., independent component analysis, have been adopted to define functional networks, which are considered more functionally homogeneous and may be better at capturing individual differences of real functional boundaries than those defined by existing atlases (Calhoun et al., 2001). Therefore, our results should be validated in further studies utilizing data‐driven parcellation approaches. Finally, since the resting‐state fMRI field‐of‐view did not include the entire cerebellum in all subjects, we only calculated functional connectivity gradients of the insula to the cerebral systems, leaving the insula‐to‐cerebellum functional connectivity gradients elusive.

In conclusion, using a combination of resting‐state fMRI and functional connectivity gradients, we established three different topographic patterns of the insula reflecting its rsFC profiles to the higher‐order transmodal associative, lower‐order unimodal primary, and subcortical systems. Apart from complementing and extending previous literature on the heterogeneous connectivity patterns of insula subregions, the presented framework may offer ample opportunities to refine our understanding of the role of the insula in many brain disorders, e.g., quantifying patient‐specific deviations from normative gradients and relating these gradients to diagnostic labels, clinical symptoms, and cognitive impairments.

## CONFLICT OF INTEREST

The authors declare no conflict of interests.

## Supporting information


Appendix S1:

## Data Availability

The data that support the findings of this study are available on request from the corresponding author. The data are not publicly available due to privacy or ethical restrictions.
