# Supplementary material for: Functional connectivity gradients of the insula to different cerebral systems
Source: Hum Brain Mapp. 2022 Oct 7;44(2):790–800. doi: 10.1002/hbm.26099 (PMC9842882; doi:10.1002/hbm.26099)
Supplement: Supplementary file 1 — Appendix S1: [file HBM-44-790-s001.doc]

**Supplementary materials**

**Table S1. Demographic information of the discovery and validation datasets**

| **Dataset** | **Sample size** | **Age (years)** | **Gender (F/M)** | **FD (mm)** |
| --- | --- | --- | --- | --- |
| Discovery | 361 | 28.84 ± 10.83 | 183/178 | 0.13 ± 0.07 |
| CNP | 103 | 30.87 ± 8.56 | 47/56 | 0.17 ± 0.08 |
| SALD | 329 | 37.81 ± 13.79 | 207/122 | 0.15 ± 0.08 |

Age and FD are expressed as mean ± standard deviation. Abbreviations: CNP, Consortium for Neuropsychiatric Phenomics; SALD, Southwest University Adult Lifespan Dataset; F, female; M, male; FD, frame-wise displacement.

**Table S2.** Resting-state functional MRI parameters for three datasets

| **Parameters** | **Discovery** | **CNP** | **SALD** |
| --- | --- | --- | --- |
| Scanner | 3.0T General Electric Discovery MR750w | 3.0T Siemens Trio | 3.0T Siemens Trio |
| Sequence | GRE-SS-EPI | T2^*^-weighted EPI | GRE-EPI |
| TR (ms) | 2000 | 2000 | 2000 |
| TE (ms) | 30 | 30 | 30 |
| FA (°) | 90 | 90 | 90 |
| FOV (mm^2^) | 220 × 220 | 192 × 192 | 220 × 220 |
| Matrix size | 64 × 64 | 64 × 64 | 64 × 64 |
| Slice thickness (mm) | 3 | 4 | 3 |
| Slice gap (mm) | 1 | - | 1 |
| Slices | 35 | 34 | 32 |
| Time points | 185 | 152 | 242 |

Abbreviations: MRI, magnetic resonance imaging; CNP, Consortium for Neuropsychiatric Phenomics; SALD, Southwest University Adult Lifespan Dataset; GRE, gradient echo; SS, single shot; EPI, echo planar imaging; TR, repetition time; TE, echo time; FA, flip angle; FOV, field of view.

**Table S3.** Ontology and nomenclature of brain areas comprising different cerebral systems in the Human Brainnetome Atlas

| **System** | **Gyrus** | **Left and right hemispheres** | **Label ID.L** | **Label ID.R** | **Modified cyto-architectonic** | **lh.MNI (*X, Y, Z*)** | **rh.MNI (*X, Y, Z*)** |
| --- | --- | --- | --- | --- | --- | --- | --- |
| Insula cortex | INS, Insular gyrus | INS_L(R)_6_1 | 163 | 164 | G, hypergranular insula | −36, −20, 10 | 37, −18, 8 |
|  |  | INS_L(R)_6_2 | 165 | 166 | vIa, ventral agranular insula | −32, 14, −13 | 33, 14, −13 |
|  |  | INS_L(R)_6_3 | 167 | 168 | dIa, dorsal agranular insula | −34, 18, 1 | 36, 18, 1 |
|  |  | INS_L(R)_6_4 | 169 | 170 | vId/vIg, ventral dysgranular and granular insula | −38, −4, −9 | 39, −2, −9 |
|  |  | INS_L(R)_6_5 | 171 | 172 | dIg, dorsal granular insula | −38, −8, 8 | 39, −7, 8 |
|  |  | INS_L(R)_6_6 | 173 | 174 | dId, dorsal dysgranular insula | −38, 5, 5 | 38, 5, 5 |
| Prefrontal cortex | SFG, Superior frontal gyrus | SFG_L(R)_7_1 | 1 | 2 | A8m, medial area 8 | −5 ,15, 54 | 7, 16, 54 |
|  |  | SFG_L(R)_7_2 | 3 | 4 | A8dl, dorsolateral area 8 | −18, 24, 53 | 22, 26, 51 |
|  |  | SFG_L(R)_7_3 | 5 | 6 | A9l, lateral area 9 | −11, 49, 40 | 13, 48, 40 |
|  |  | SFG_L(R)_7_4 | 7 | 8 | A6dl, dorsolateral area 6 | −18, −1, 65 | 20, 4, 64 |
|  |  | SFG_L(R)_7_5 | 9 | 10 | A6m, medial area 6 | −6, −5, 58 | 7, −4, 60 |
|  |  | SFG_L(R)_7_6 | 11 | 12 | A9m, medial area 9 | −5, 36, 38 | 6, 38, 35 |
|  |  | SFG_L(R)_7_7 | 13 | 14 | A10m, medial area 10 | −8, 56, 15 | 8, 58, 13 |
|  | MFG, Middle frontal gyrus | MFG_L(R)_7_1 | 15 | 16 | A9/46d, dorsal area 9/46 | −27, 43, 31 | 30, 37, 36 |
|  |  | MFG_L(R)_7_2 | 17 | 18 | IFJ, inferior frontal junction | −42, 13, 36 | 42, 11, 39 |
|  |  | MFG_L(R)_7_3 | 19 | 20 | A46, area 46 | −28, 56, 12 | 28, 55, 17 |
|  |  | MFG_L(R)_7_4 | 21 | 22 | A9/46v, ventral area 9/46 | −41, 41, 16 | 42, 44, 14 |
|  |  | MFG_L(R)_7_5 | 23 | 24 | A8vl, ventrolateral area 8 | −33, 23, 45 | 42, 27, 39 |
|  |  | MFG_L(R)_7_6 | 25 | 26 | A6vl, ventrolateral area 6 | −32, 4, 55 | 34, 8, 54 |
|  |  | MFG_L(R)_7_7 | 27 | 28 | A10l, lateral area 10 | −26, 60, −6 | 25, 61, −4 |
|  | IFG, Inferior frontal gyrus | IFG_L(R)_6_1 | 29 | 30 | A44d, dorsal area 44 | −46, 13, 24 | 45, 16, 25 |
|  |  | IFG_L(R)_6_2 | 31 | 32 | IFS, inferior frontal sulcus | −47, 32, 14 | 48, 35, 13 |
|  |  | IFG_L(R)_6_3 | 33 | 34 | A45c, caudal area 45 | −53, 23, 11 | 54, 24, 12 |
|  |  | IFG_L(R)_6_4 | 35 | 36 | A45r, rostral area 45 | −49, 36, −3 | 51, 36, −1 |
|  |  | IFG_L(R)_6_5 | 37 | 38 | A44op, opercular area 44 | −39, 23, 4 | 42, 22, 3 |
|  |  | IFG_L(R)_6_6 | 39 | 40 | A44v, ventral area 44 | −52, 13, 6 | 54, 14, 11 |
|  | OrG, Orbital gyrus | OrG_L(R)_6_1 | 41 | 42 | A14m, medial area 14 | −7, 54, −7 | 6, 47, −7 |
|  |  | OrG_L(R)_6_2 | 43 | 44 | A12/47o, orbital area 12/47 | −36, 33, −16 | 40, 39, −14 |
|  |  | OrG_L(R)_6_3 | 45 | 46 | A11l, lateral area 11 | −23, 38, −18 | 23, 36, −18 |
|  |  | OrG_L(R)_6_4 | 47 | 48 | A11m, medial area 11 | −6, 52, −19 | 6, 57, −16 |
|  |  | OrG_L(R)_6_5 | 49 | 50 | A13, area 13 | −10, 18, −19 | 9, 20, −19 |
|  |  | OrG_L(R)_6_6 | 51 | 52 | A12/47l, lateral area 12/47 | −41, 32, −9 | 42, 31, −9 |
| Motor cortex | PrG, Precentral gyrus | PrG_L(R)_6_1 | 53 | 54 | A4hf, area 4 (head and face region) | −49, −8, 39 | 55, −2, 33 |
|  |  | PrG_L(R)_6_2 | 55 | 56 | A6cdl, caudal dorsolateral area 6 | −32, −9, 58 | 33, −7, 57 |
|  |  | PrG_L(R)_6_3 | 57 | 58 | A4ul, area 4 (upper limb region) | −26, −25, 63 | 34, −19, 59 |
|  |  | PrG_L(R)_6_4 | 59 | 60 | A4t, area 4 (trunk region) | −13, −20, 73 | 15, −22, 71 |
|  |  | PrG_L(R)_6_5 | 61 | 62 | A4tl, area 4 (tongue and larynx region) | −52, 0, 8 | 54, 4, 9 |
|  |  | PrG_L(R)_6_6 | 63 | 64 | A6cvl, caudal ventrolateral area 6 | −49, 5, 30 | 51, 7, 30 |
|  | PCL, Paracentral lobule | PCL_L(R)_2_2 | 67 | 68 | A4ll, area 4 (lower limb region) | −4, −23, 61 | 5, −21, 61 |
| Somatosensory cortex | PCL, Paracentral lobule | PCL_L(R)_2_1 | 65 | 66 | A1/2/3ll, area 1/2/3 (lower limb region) | −8, −38, 58 | 10, −34, 54 |
|  | PoG, Postcentral gyrus | PoG_L(R)_4_1 | 155 | 156 | A1/2/3ulhf, area 1/2/3 (upper limb, head and face region) | −50, −16, 43 | 50, −14, 44 |
|  |  | PoG_L(R)_4_2 | 157 | 158 | A1/2/3tonIa, area 1/2/3 (tongue and larynx region) | −56, −14, 16 | 56, −10, 15 |
|  |  | PoG_L(R)_4_3 | 159 | 160 | A2, area 2 | −46, −30, 50 | 48, −24, 48 |
|  |  | PoG_L(R)_4_4 | 161 | 162 | A1/2/3tru, area 1/2/3 (trunk region) | −21, −35, 68 | 20, −33, 69 |
| Posterior parietal cortex | SPL, Superior parietal lobule | SPL_L(R)_5_1 | 125 | 126 | A7r, rostral area 7 | −16, −60, 63 | 19, −57, 65 |
|  |  | SPL_L(R)_5_2 | 127 | 128 | A7c, caudal area 7 | −15, −71, 52 | 19, −69, 54 |
|  |  | SPL_L(R)_5_3 | 129 | 130 | A5l, lateral area 5 | −33, −47, 50 | 35, −42, 54 |
|  |  | SPL_L(R)_5_4 | 131 | 132 | A7pc, postcentral area 7 | −22, −47, 65 | 23, −43, 67 |
|  |  | SPL_L(R)_5_5 | 133 | 134 | A7ip, intraparietal area 7 (hIP3) | −27, −59, 54 | 31, −54, 53 |
|  | IPL, Inferior parietal lobule | IPL_L(R)_6_1 | 135 | 136 | A39c, caudal area 39 (PGp) | −34, −80, 29 | 45, −71, 20 |
|  |  | IPL_L(R)_6_2 | 137 | 138 | A39rd, rostrodorsal area 39 (Hip3) | −38, −61, 46 | 39, −65, 44 |
|  |  | IPL_L(R)_6_3 | 139 | 140 | A40rd, rostrodorsal area 40 (PFt) | −51, −33, 42 | 47, −35, 45 |
|  |  | IPL_L(R)_6_4 | 141 | 142 | A40c, caudal area 40 (PFm) | −56, −49, 38 | 57, −44, 38 |
|  |  | IPL_L(R)_6_5 | 143 | 144 | A39rv, rostroventral area 39 (PGa) | −47, −65, 26 | 53, −54, 25 |
|  |  | IPL_L(R)_6_6 | 145 | 146 | A40rv, rostroventral area 40 (PFop) | −53, −31, 23 | 55, −26, 26 |
|  | Pcun, Precuneus | PCun_L(R)_4_1 | 147 | 148 | A7m, medial area 7 (PEp) | −5, −63, 51 | 6, −65, 51 |
|  |  | PCun_L(R)_4_2 | 149 | 150 | A5m, medial area 5 (PEm) | −8, −47, 57 | 7, −47, 58 |
|  |  | PCun_L(R)_4_3 | 151 | 152 | dmPOS, dorsomedial parietooccipital sulcus (PEr) | −12, −67, 25 | 16, −64, 25 |
|  |  | PCun_L(R)_4_4 | 153 | 154 | A31, area 31 (Lc1) | −6, −55, 34 | 6, −54, 35 |
| Occipital cortex | MVOcC, MedioVentral occipital cortex | MVOcC _L(R)_5_1 | 189 | 190 | cLinG, caudal lingual gyrus | −11, −82, −11 | 10, −85, −9 |
|  |  | MVOcC _L(R)_5_2 | 191 | 192 | rCunG, rostral cuneus gyrus | −5, −81, 10 | 7, −76, 11 |
|  |  | MVOcC _L(R)_5_3 | 193 | 194 | cCunG, caudal cuneus gyrus | −6, −94, 1 | 8, −90, 12 |
|  |  | MVOcC _L(R)_5_4 | 195 | 196 | rLinG, rostral lingual gyrus | −17, −60, −6 | 18, −60, −7 |
|  |  | MVOcC _L(R)_5_5 | 197 | 198 | vmPOS,ventromedial parietooccipital sulcus | −13, −68, 12 | 15, −63, 12 |
|  | LOcC, Lateral occipital cortex | LOcC_L(R)_4_1 | 199 | 200 | mOccG, middle occipital gyrus | −31, −89, 11 | 34, −86, 11 |
|  |  | LOcC _L(R)_4_2 | 201 | 202 | V5/MT+, area V5/MT+ | −46, −74, 3 | 48, −70, −1 |
|  |  | LOcC _L(R)_4_3 | 203 | 204 | OPC, occipital polar cortex | −18, −99, 2 | 22, −97, 4 |
|  |  | LOcC_L(R)_4_4 | 205 | 206 | iOccG, inferior occipital gyrus | −30, −88, −12 | 32, −85, −12 |
|  |  | LOcC_L(R)_2_1 | 207 | 208 | msOccG, medial superior occipital gyrus | −11, −88, 31 | 16, −85, 34 |
|  |  | LOcC_L(R)_2_2 | 209 | 210 | lsOccG, lateral superior occipital gyrus | −22, −77, 36 | 29, −75, 36 |
| Temporal cortex | STG, Superior temporal gyrus | STG_L(R)_6_1 | 69 | 70 | A38m, medial area 38 | −32, 14, −34 | 31, 15, −34 |
|  |  | STG_L(R)_6_2 | 71 | 72 | A41/42, area 41/42 | −54, −32, 12 | 54, −24, 11 |
|  |  | STG_L(R)_6_3 | 73 | 74 | TE1.0 and TE1.2 | −50, −11, 1 | 51, −4, −1 |
|  |  | STG_L(R)_6_4 | 75 | 76 | A22c, caudal area 22 | −62, −33, 7 | 66, −20, 6 |
|  |  | STG_L(R)_6_5 | 77 | 78 | A38l, lateral area 38 | −45, 11, −20 | 47, 12, −20 |
|  |  | STG_L(R)_6_6 | 79 | 80 | A22r, rostral area 22 | −55, −3, −10 | 56, −12, −5 |
|  | MTG, Middle temporal gyrus | MTG_L(R)_4_1 | 81 | 82 | A21c, caudal area 21 | −65, −30, −12 | 65, −29, −13 |
|  |  | MTG_L(R)_4_2 | 83 | 84 | A21r, rostral area 21 | −53, 2, −30 | 51, 6, −32 |
|  |  | MTG_L(R)_4_3 | 85 | 86 | A37dl, dorsolateral area 37 | −59, −58, 4 | 60, −53, 3 |
|  |  | MTG_L(R)_4_4 | 87 | 88 | aSTS, anterior superior temporal sulcus | −58, −20, −9 | 58, −16, −10 |
|  | ITG, Inferior temporal gyrus | ITG_L(R)_7_1 | 89 | 90 | A20iv, intermediate ventral area 20 | −45, −26, −27 | 46, −14, −33 |
|  |  | ITG_L(R)_7_2 | 91 | 92 | A37elv, extreme lateroventral area 37 | −51, −57, −15 | 53, −52, −18 |
|  |  | ITG_L(R)_7_3 | 93 | 94 | A20r, rostral area 20 | −43, −2, −41 | 40, 0, −43 |
|  |  | ITG_L(R)_7_4 | 95 | 96 | A20il, intermediate lateral area 20 | −56, −16, −28 | 55, −11, −32 |
|  |  | ITG_L(R)_7_5 | 97 | 98 | A37vl, ventrolateral area 37 | −55, −60, −6 | 54, −57, −8 |
|  |  | ITG_L(R)_7_6 | 99 | 100 | A20cl, caudolateral of area 20 | −59, −42, −16 | 61, −40, −17 |
|  |  | ITG_L(R)_7_7 | 101 | 102 | A20cv, caudoventral of area 20 | −55, −31, −27 | 54, −31, −26 |
|  | FuG, Fusiform gyrus | FuG_L(R)_3_1 | 103 | 104 | A20rv, rostroventral area 20 | −33, −16, −32 | 33, −15, −34 |
|  |  | FuG_L(R)_3_2 | 105 | 106 | A37mv, medioventral area 37 | −31, −64, −14 | 31, −62, −14 |
|  |  | FuG_L(R)_3_3 | 107 | 108 | A37lv, lateroventral area 37 | −42, −51, −17 | 43, −49, −19 |
|  | PhG, Parahippocampal gyrus | PhG_L(R)_6_1 | 109 | 110 | A35/36r, rostral area 35/36 | −27, −7, −34 | 28, −8, −33 |
|  |  | PhG_L(R)_6_2 | 111 | 112 | A35/36c, caudal area 35/36 | −25, −25, −26 | 26, −23, −27 |
|  |  | PhG_L(R)_6_3 | 113 | 114 | TL, area TL (lateral PPHC, posterior parahippocampal gyrus) | −28, −32, −18 | 30, −30, −18 |
|  |  | PhG_L(R)_6_4 | 115 | 116 | A28/34, area 28/34 (EC, entorhinal cortex) | −19, −12, −30 | 19, −10, −30 |
|  |  | PhG_L(R)_6_5 | 117 | 118 | TI, area TI (temporal agranular insular cortex) | −23, 2, −32 | 22, 1, −36 |
|  |  | PhG_L(R)_6_6 | 119 | 120 | TH, area TH (medial PPHC) | −17, −39, −10 | 19, −36, −11 |
|  | pSTS, Posterior superior temporal sulcus | pSTS_L(R)_2_1 | 121 | 122 | rpSTS, rostroposterior superior temporal sulcus | −54, −40, 4 | 53, −37, 3 |
|  |  | pSTS_L(R)_2_2 | 123 | 124 | cpSTS, caudoposterior superior temporal sulcus | −52, −50, 11 | 57, −40, 12 |
| Limbic lobule | CG, Cingulate gyrus | CG_L(R)_7_1 | 175 | 176 | A23d, dorsal area 23 | −4, −39, 31 | 4, −37, 32 |
|  |  | CG_L(R)_7_2 | 177 | 178 | A24rv, rostroventral area 24 | −3, 8, 25 | 5, 22, 12 |
|  |  | CG_L(R)_7_3 | 179 | 180 | A32p, pregenual area 32 | −6, 34, 21 | 5, 28, 27 |
|  |  | CG_L(R)_7_4 | 181 | 182 | A23v, ventral area 23 | −8, −47, 10 | 9, −44, 11 |
|  |  | CG_L(R)_7_5 | 183 | 184 | A24cd, caudodorsal area 24 | −5, 7, 37 | 4, 6, 38 |
|  |  | CG_L(R)_7_6 | 185 | 186 | A23c, caudal area 24 | −7, −23, 41 | 6, −20, 40 |
|  |  | CG_L(R)_7_7 | 187 | 188 | A32sg, subgenual area 32 | −4, 39, −2 | 5, 41, 6 |
| Subcortical nuclei | Amyg, Amygdala | Amyg_L(R)_2_1 | 211 | 212 | mAmyg, medial amygdala | −19, −2, −20 | 19, −2, −19 |
|  |  | Amyg_L(R)_2_2 | 213 | 214 | lAmyg, lateral amygdala | −27, −4, −20 | 28, −3, −20 |
|  | Hipp, Hippocampus | Hipp_L(R)_2_1 | 215 | 216 | rHipp, rostral hippocampus | −22, −14, −19 | 22, −12, −20 |
|  |  | Hipp_L(R)_2_2 | 217 | 218 | cHipp, caudal hippocampus | −28, −30, −10 | 29, −27, −10 |
|  | BG, Basal ganglia | BG_L(R)_6_1 | 219 | 220 | vCa, ventral caudate | −12, 14, 0 | 15, 14, −2 |
|  |  | BG_L(R)_6_2 | 221 | 222 | GP, globus pallidus | −22, −2, 4 | 22, −2, 3 |
|  |  | BG_L(R)_6_3 | 223 | 224 | NAC, nucleus accumbens | −17, 3, −9 | 15, 8, −9 |
|  |  | BG_L(R)_6_4 | 225 | 226 | vmPu, ventromedial putamen | −23, 7, −4 | 22, 8, −1 |
|  |  | BG_L(R)_6_5 | 227 | 228 | dCa, dorsal caudate | −14, 2, 16 | 14, 5, 14 |
|  |  | BG_L(R)_6_6 | 229 | 230 | dlPu, dorsolateral putamen | −28, −5, 2 | 29, −3, 1 |
|  | Tha, Thalamus | Tha_L(R)_8_1 | 231 | 232 | mPFtha, medial pre-frontal thalamus | −7, −12, 5 | 7, −11, 6 |
|  |  | Tha_L(R)_8_2 | 233 | 234 | mPMtha, pre-motor thalamus | −18, −13, 3 | 12, −14, 1 |
|  |  | Tha_L(R)_8_3 | 235 | 236 | Stha, sensory thalamus | −18, −23, 4 | 18, −22, 3 |
|  |  | Tha_L(R)_8_4 | 237 | 238 | rTtha, rostral temporal thalamus | −7, −14, 7 | 3, −13, 5 |
|  |  | Tha_L(R)_8_5 | 239 | 240 | PPtha, posterior parietal thalamus | −16, −24, 6 | 15, −25, 6 |
|  |  | Tha_L(R)_8_6 | 241 | 242 | Otha, occipital thalamus | −15, −28, 4 | 13, −27, 8 |
|  |  | Tha_L(R)_8_7 | 243 | 244 | cTtha, caudal temporal thalamus | −12, −22, 13 | 10, −14, 14 |
|  |  | Tha_L(R)_8_8 | 245 | 246 | lPFtha, lateral pre-frontal thalamus | −11, −14, 2 | 13, −16, 7 |

Abbreviations: MNI, Montreal Neurological Institute.


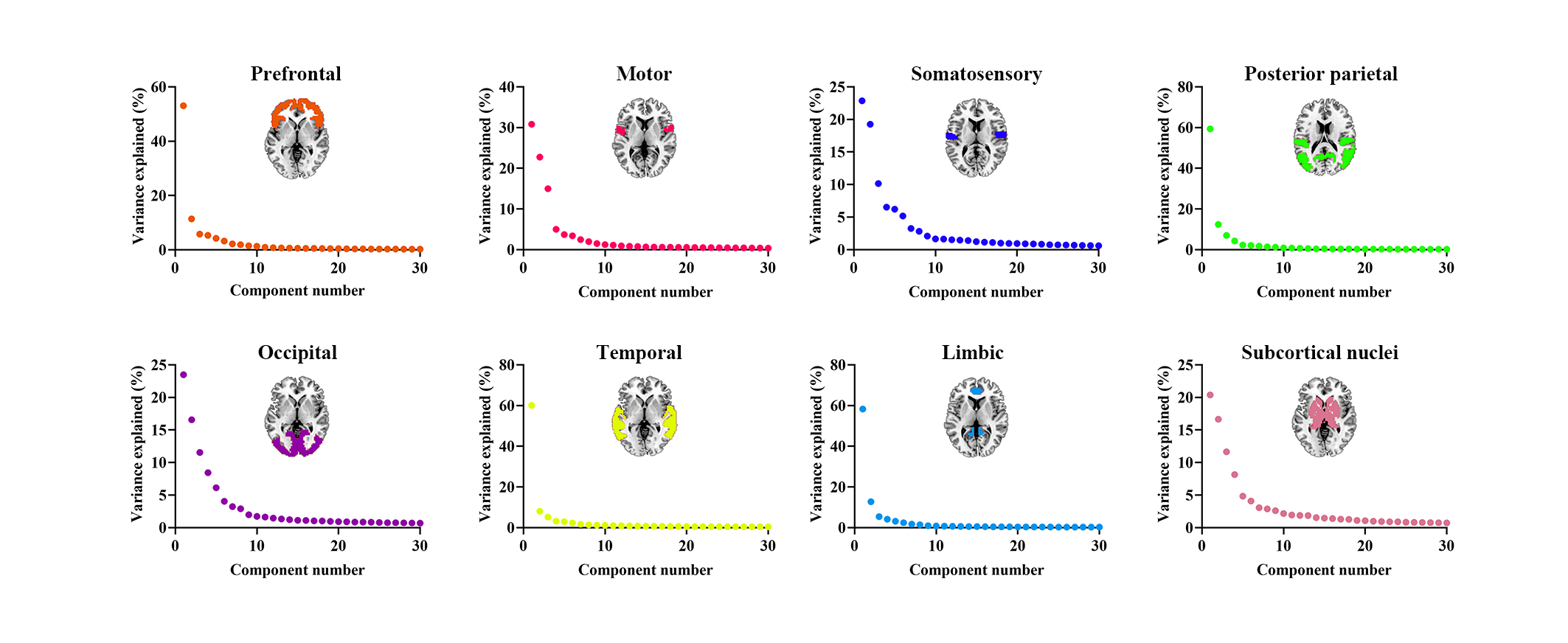


**Figure S1.** Scree plots showing the connectivity variance explained by the gradients of the insula to the eight cerebral systems.


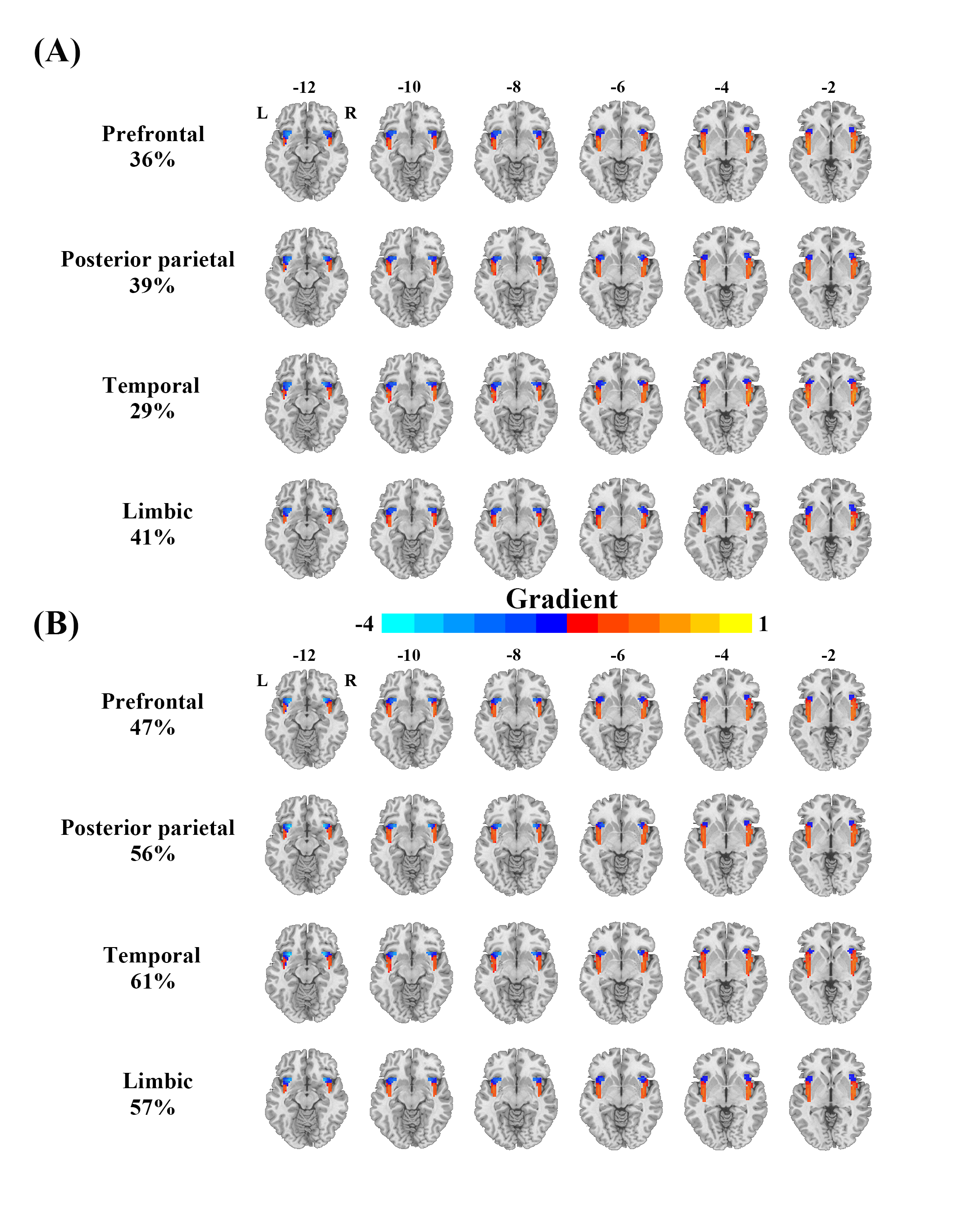


**Figure S2.** Functional connectivity gradients of the insula to the higher-order transmodal associative systems including the prefrontal, posterior parietal, temporal cortices and limbic lobule derived from the CNP (A) and SALD (B) datasets. The percentages represent connectivity variance explained by the corresponding gradients. Abbreviations: L, left; R, right; CNP, Consortium for Neuropsychiatric Phenomics; SALD: Southwest University Adult Lifespan Dataset.


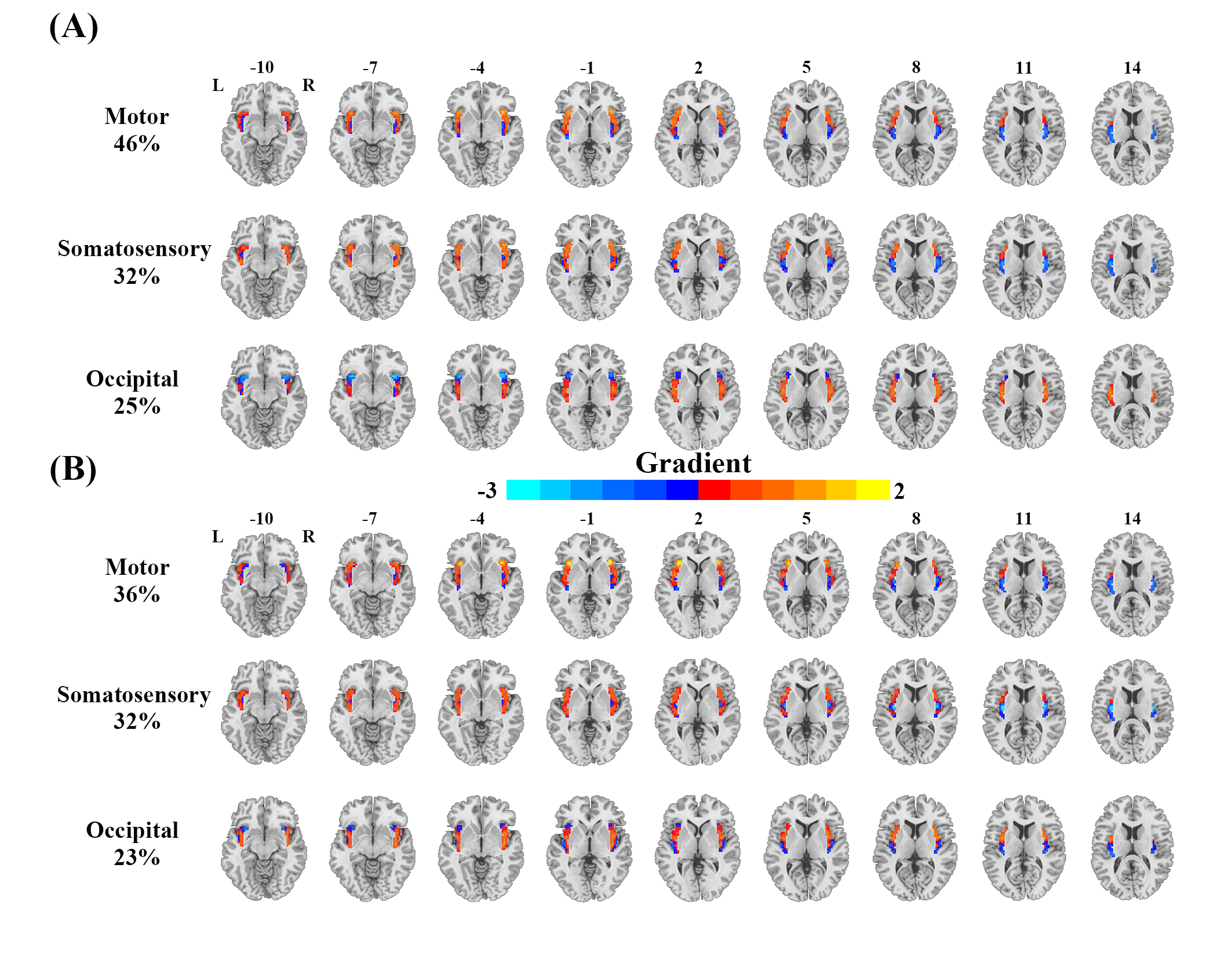


**Figure S3.** Functional connectivity gradients of the insula to the lower-order unimodal primary systems including the motor, somatosensory and occipital cortices derived from the CNP (A) and SALD (B) datasets. The percentages represent connectivity variance explained by the corresponding gradients. Abbreviations: L, left; R, right; CNP, Consortium for Neuropsychiatric Phenomics; SALD: Southwest University Adult Lifespan Dataset.


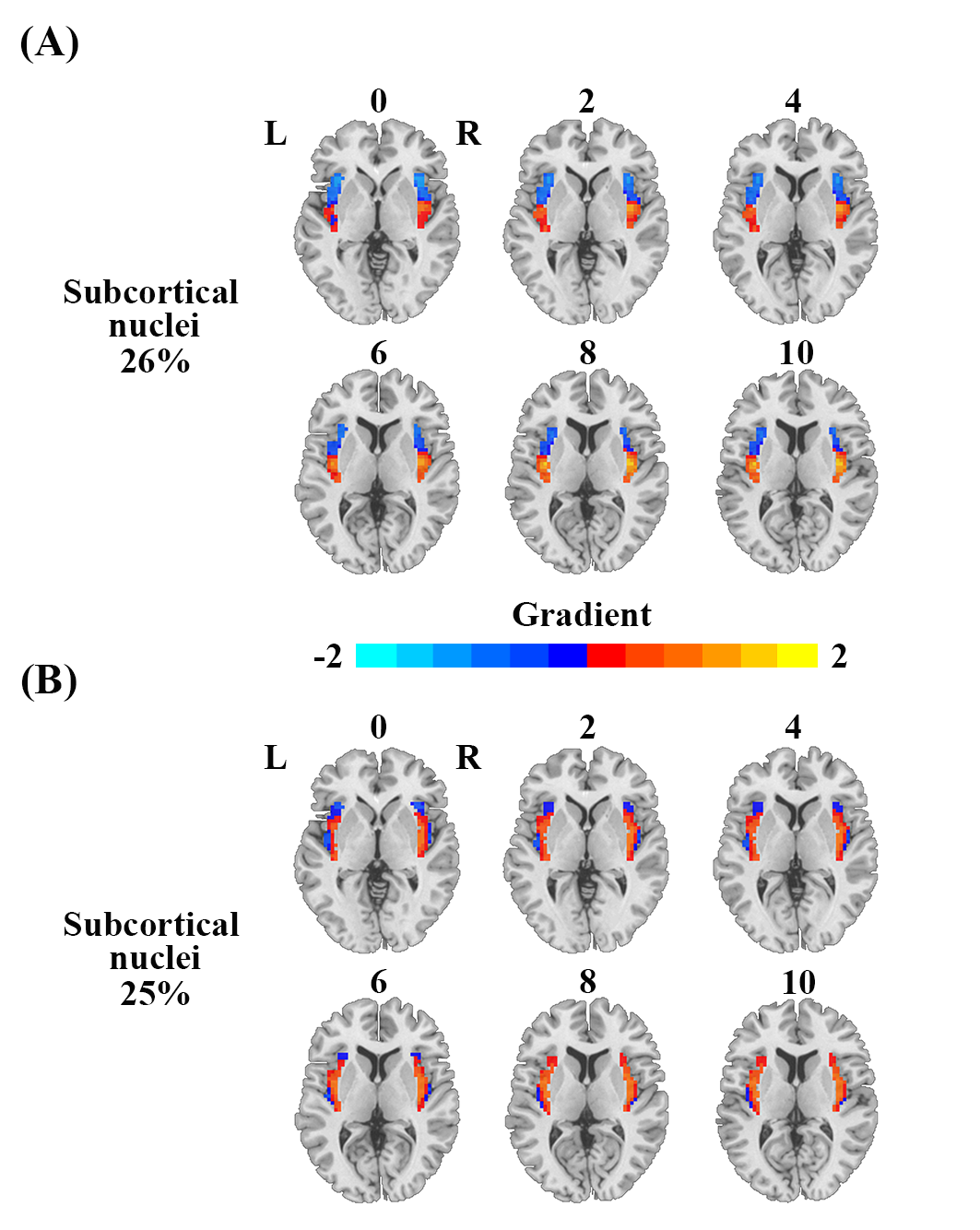


**Figure S4.** Functional connectivity gradient of the insula to the subcortical nuclei derived from the CNP (A) and SALD (B) datasets. The percentages represent connectivity variance explained by the corresponding gradients. Abbreviations: L, left; R, right; CNP, Consortium for Neuropsychiatric Phenomics; SALD: Southwest University Adult Lifespan Dataset.


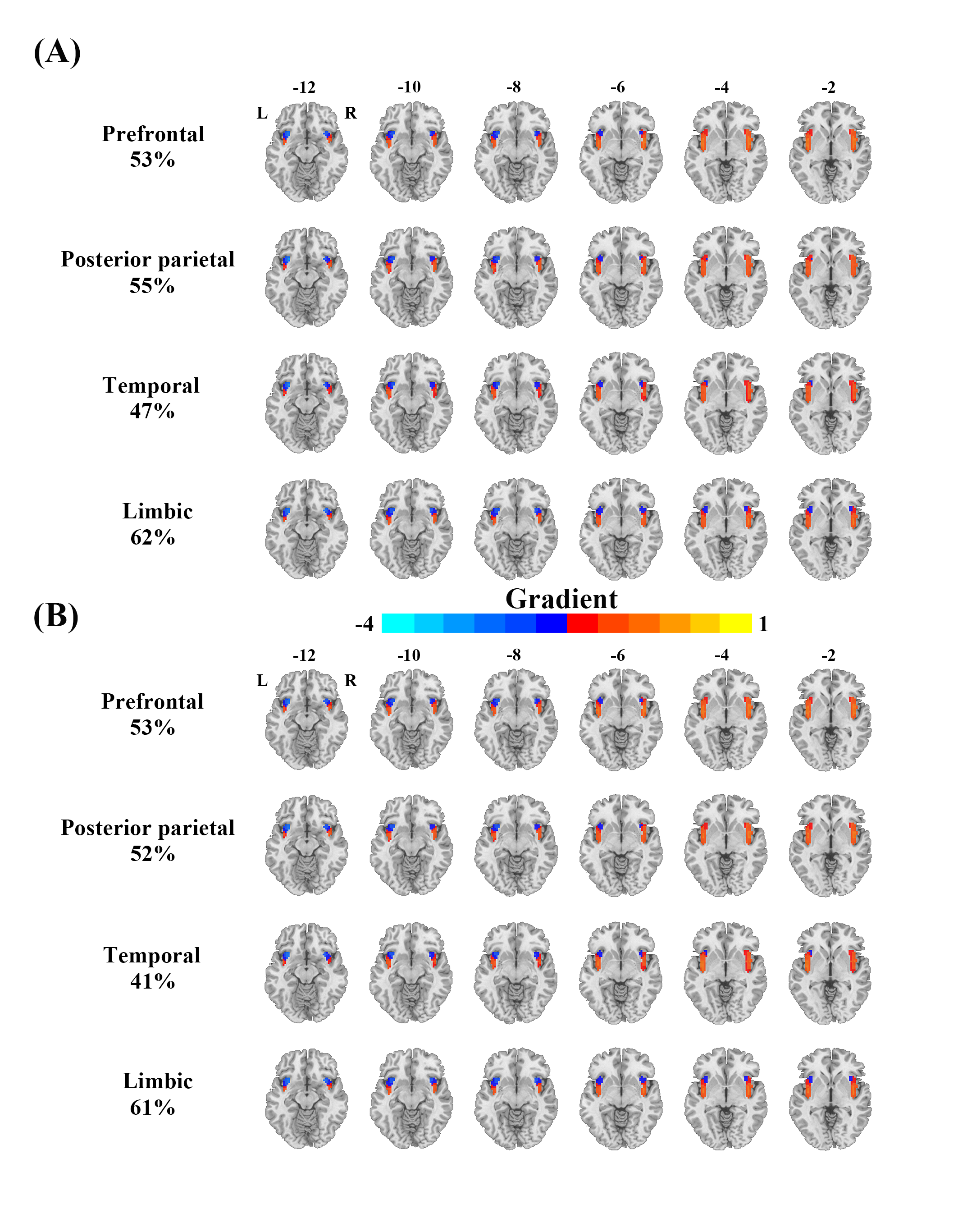


**Figure S5.** Functional connectivity gradients of the insula to the higher-order transmodal associative systems including the prefrontal, posterior parietal, temporal cortices and limbic lobule derived from two other rsFC matrix thresholds of top 20% (A) and 30% (B). The percentages represent connectivity variance explained by the corresponding gradients. Abbreviations: L, left; R, right; rsFC, resting-state functional connectivity.


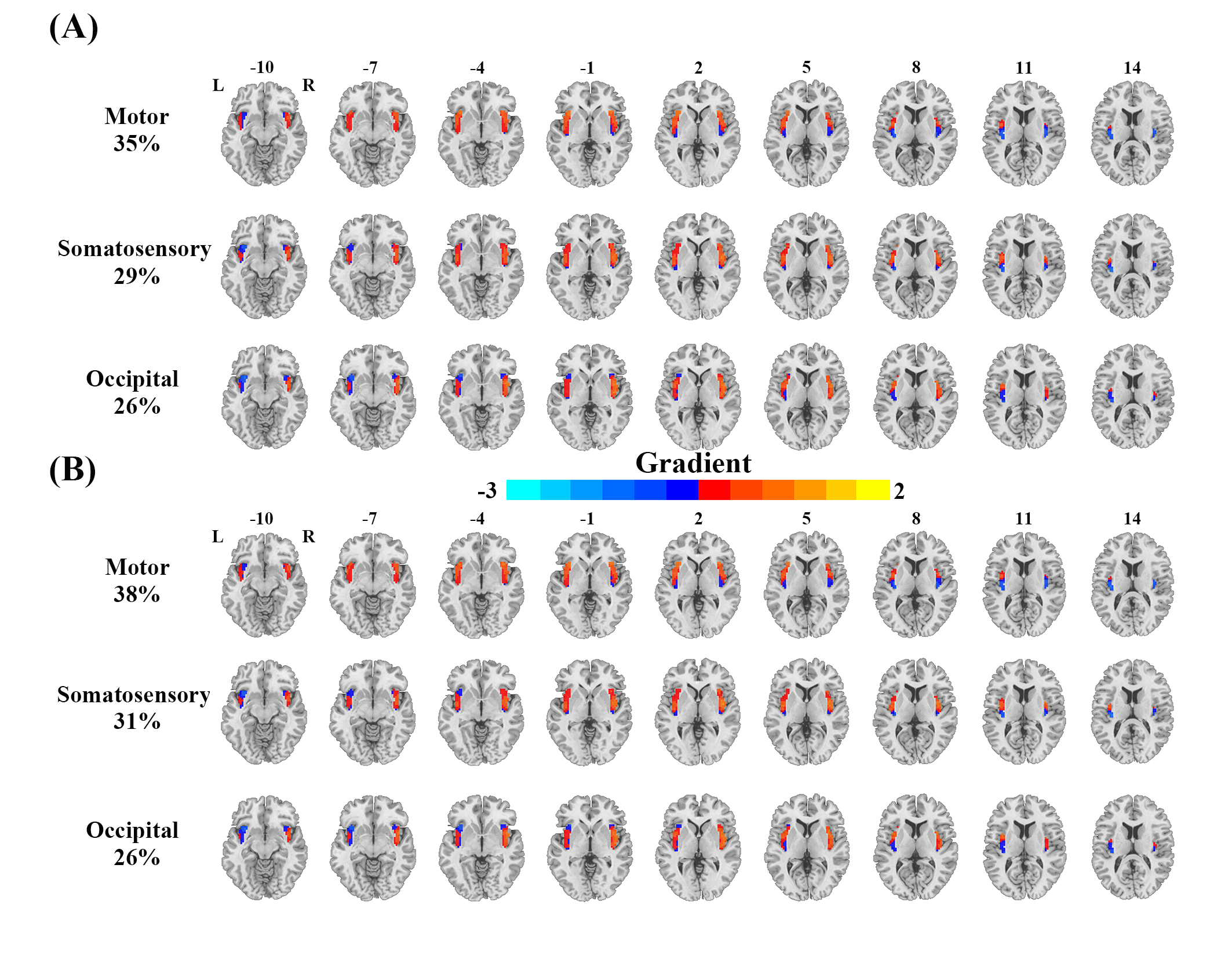


**Figure S6.** Functional connectivity gradients of the insula to the lower-order unimodal primary systems including the motor, somatosensory and occipital cortices derived from two other rsFC matrix thresholds of top 20% (A) and 30% (B). The percentages represent connectivity variance explained by the corresponding gradients. Abbreviations: L, left; R, right; rsFC, resting-state functional connectivity.


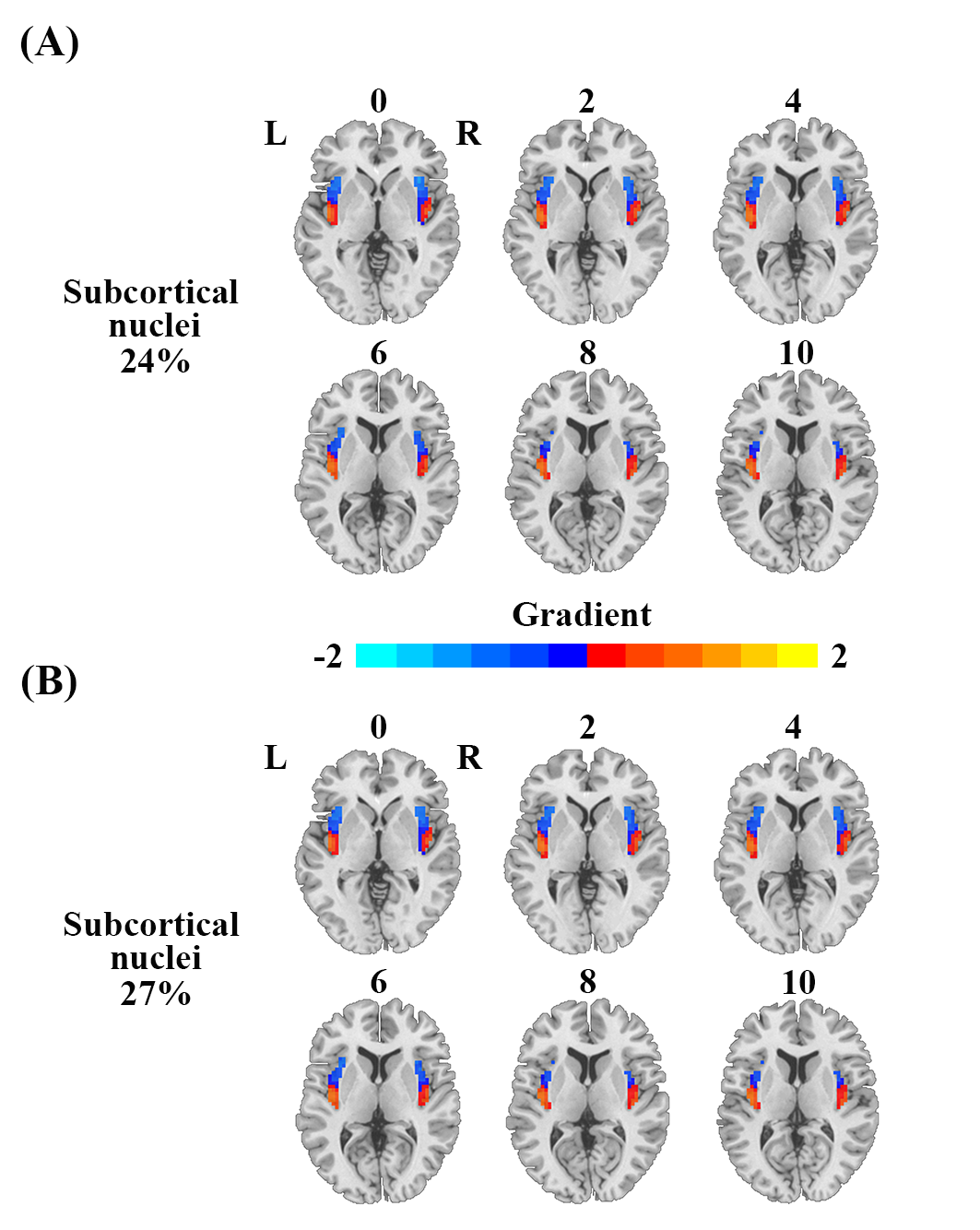


**Figure S7.** Functional connectivity gradient of the insula to the subcortical nuclei derived from two other rsFC matrix thresholds of top 20% (A) and 30% (B). The percentages represent connectivity variance explained by the corresponding gradients. Abbreviations: L, left; R, right; rsFC, resting-state functional connectivity.


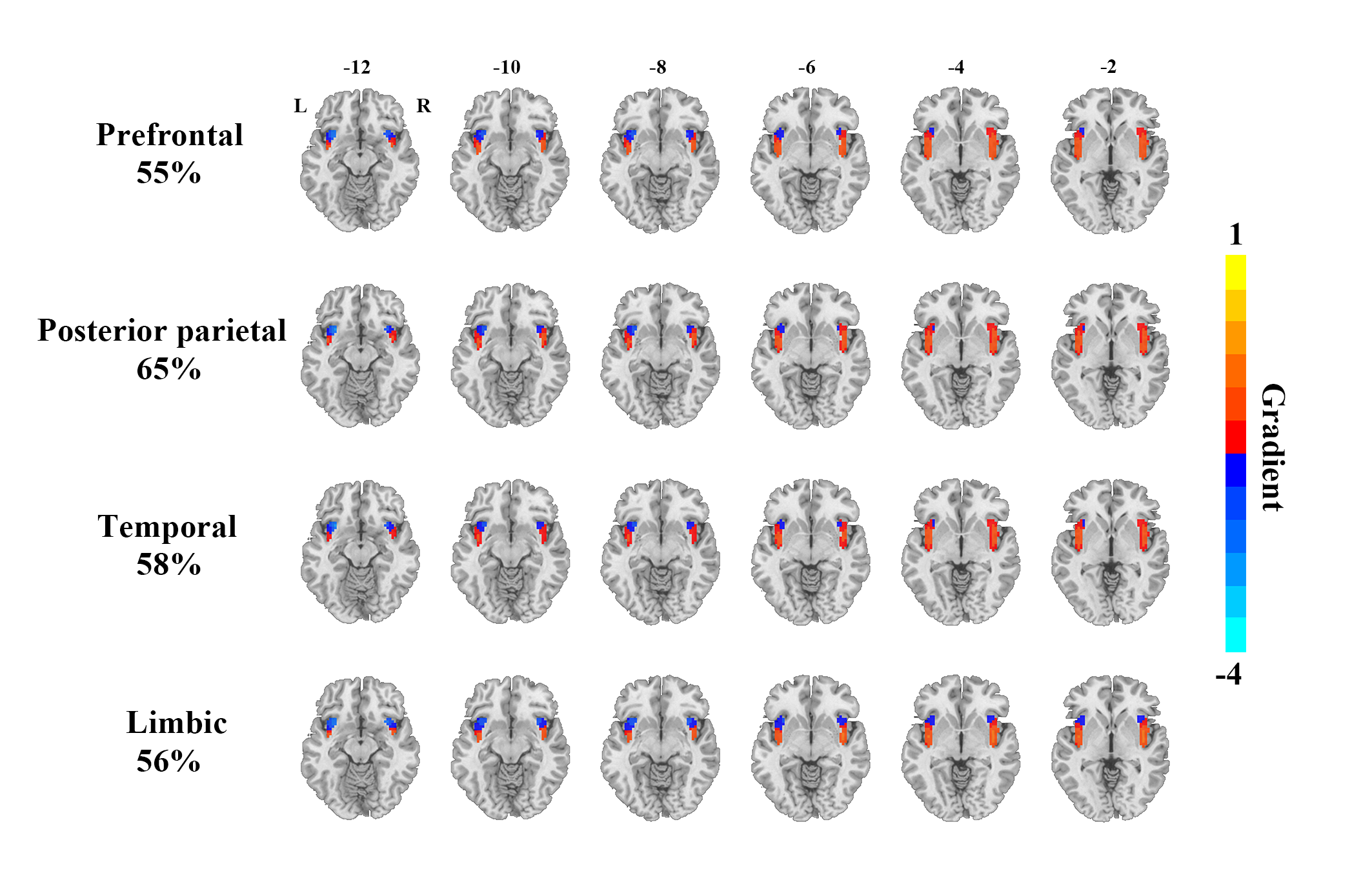


**Figure S8.** Functional connectivity gradients of the insula to the higher-order transmodal associative systems including the prefrontal, posterior parietal, temporal cortices and limbic lobule derived from BOLD data with GSR. The percentages represent connectivity variance explained by the corresponding gradients. Abbreviations: L, left; R, right; BOLD, blood-oxygen-level-dependent; GSR, global signal regression.


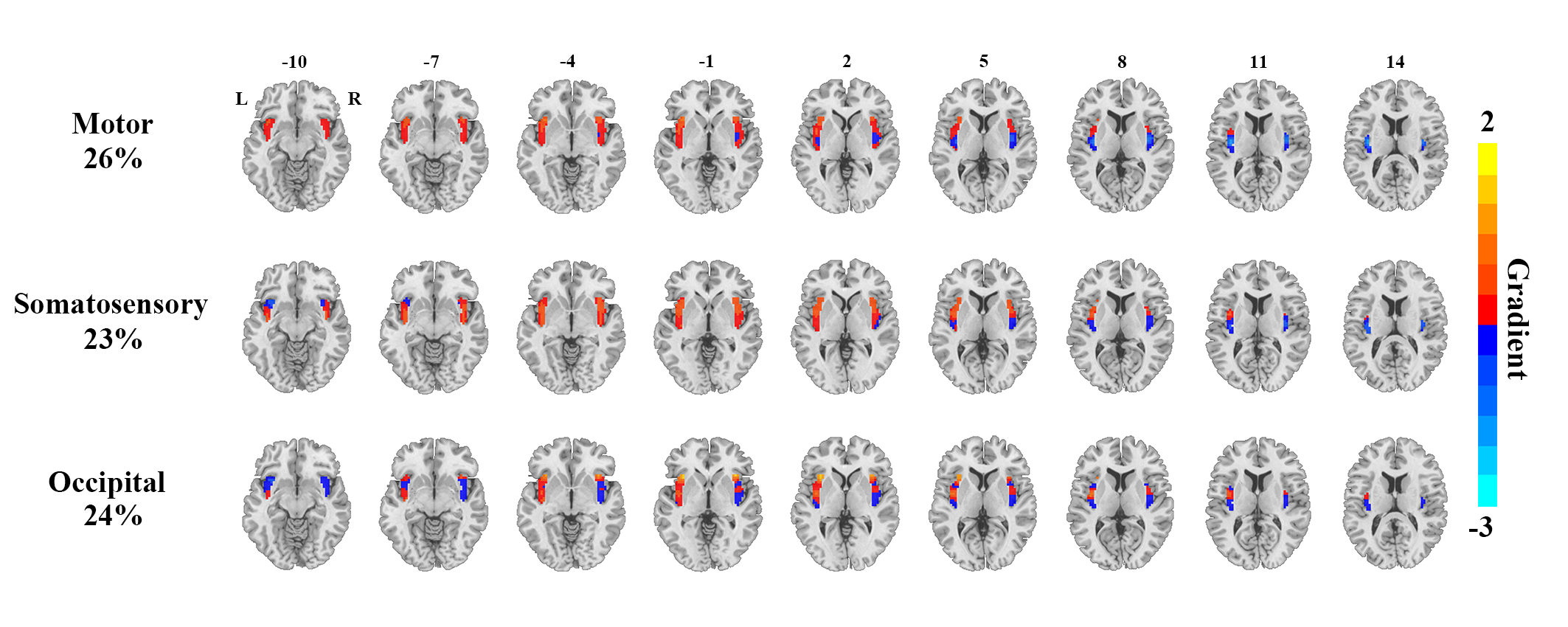


**Figure S9.** Functional connectivity gradients of the insula to the lower-order unimodal primary systems including the motor, somatosensory and occipital cortices derived from BOLD data with GSR. The percentages represent connectivity variance explained by the corresponding gradients. Abbreviations: L, left; R, right; BOLD, blood-oxygen-level-dependent; GSR, global signal regression.


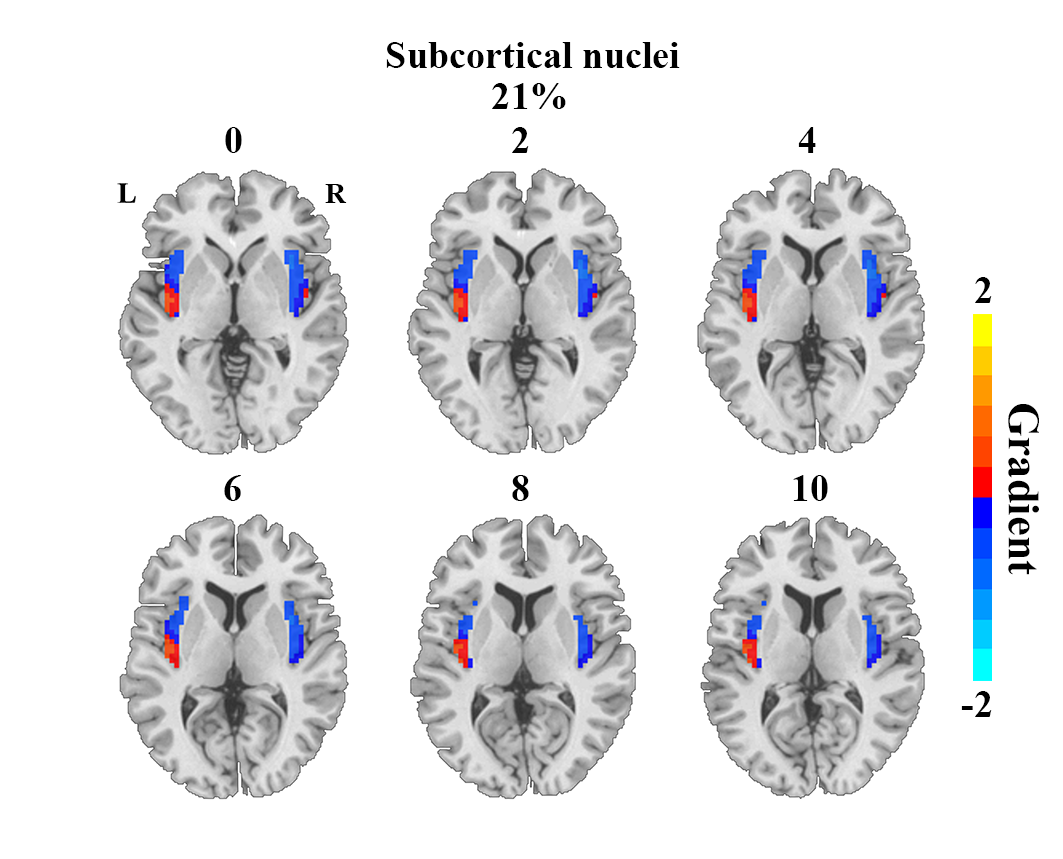


**Figure S10.** Functional connectivity gradient of the insula to the subcortical nuclei derived from BOLD data with GSR. The percentage represents connectivity variance explained by the gradient. Abbreviations: L, left; R, right; BOLD, blood-oxygen-level-dependent; GSR, global signal regression.
